# Prevalence and influencing factors of probiotic usage among colorectal cancer patients in China: A national database study

**DOI:** 10.1371/journal.pone.0291864

**Published:** 2023-09-21

**Authors:** Difei Yao, Wei He, Yangmin Hu, Ying Yuan, Huimin Xu, Juan Wang, Haibin Dai

**Affiliations:** 1 Department of Pharmacy, Second Affiliated Hospital of Zhejiang University School of Medicine, Hangzhou, Zhejiang, China; 2 Research Center for Clinical Pharmacy, Zhejiang University, Hangzhou, Zhejiang, China; 3 Department of Medical Oncology, Second Affiliated Hospital of Zhejiang University School of Medicine, Hangzhou, Zhejiang, China; 4 Key Laboratory of Cancer Prevention and Intervention, Cancer Institute, Ministry of Education, Second Affiliated Hospital of Zhejiang University School of Medicine, Hangzhou, Zhejiang, China; Universidad San Francisco de Quito, ECUADOR

## Abstract

Probiotics have become increasingly popular among cancer patients. However, there is limited data from a real-world setting. This study aims to conduct a retrospective analysis to understand the trend of probiotic prescriptions in Chinese colorectal cancer patients. The Mann-Kendall and Cochran-Armitage trend test was applied to estimate the trend significance. Gephi software identified the combination of probiotic strains. The binary logistic regression investigated influence factors, and Spearman’s rank correlation coefficient calculated correlations between probiotics and antitumor drug usage. The probiotic prescription percentage increased from 3.3% in 2015 to 4.2% in 2021 (Z = 12.77, *p* < 0.001). Although 48.3% of probiotic prescriptions had no indication-related diagnosis, diarrhea (OR 10.91, 95% CI 10.57–11.26) and dyspepsia (3.97, 3.82–4.12) included prescriptions most likely to contain probiotics. Prescriptions from the tertiary hospital (1.43,1.36–1.50), clinics (1.30, 1.28–1.33), and senior patients (1.018 per year, 1.017–1.019) were more likely to contain probiotics. Most probiotic prescriptions (95.0%) contained one probiotic product but multiple strains (69.3%). *Enterococcus faecalis* (49.7%), *Lactobacillus acidophilus* (39.4%), and *Clostridium butyricum* (27.9%) were the most prescribed strains. The probiotics co-prescribed with antitumor agents increased rapidly from 6.6% to 13.8% in seven years (Z = 15.31, *p* < 0.001). Oral fluorouracil agents (2.35, 2.14–2.59), regorafenib (1.70,1.27–2.26), and irinotecan (1.27,1.15–1.41) had a higher probability to co-prescribed with probiotics. There was no correlation between probiotic strain selection and specific antitumor drug use. The increasing prescription of probiotics in colorectal cancer patients in China may be related to treating the gastrointestinal toxicity of anti-cancer drugs. With unapproved indications and a lack of strain selectivity, evidence-based guidelines are urgently needed to improve probiotic use in this population.

## Introduction

Colorectal cancer (CRC) is one of the most common malignant tumours worldwide, accounting for the third-highest incidence (10.0%) and second-highest (9.4%) mortality [1, 2]. CRC incidence rates tend to rise uniformly with socioeconomic development [3]. Compared with transitioning countries, incidence and mortality rates in transitioned countries are approximately 4-fold and 2.6-fold higher [1]. The trends of CRC incidence and mortality in transitioned countries (the United States, Japan, and France) are stabilizing or decreasing. However, the incidence and mortality of CRC patients in China have increased in the last decade [4, 5]. According to the estimates from GLOBOCAN (https://gco.iarc.fr/), there were 555477 new CRC patients and 286162 CRC-related deaths in China in 2020, accounting for 28.8% and 30.6% of the global total [1]. In 2020, the incidence and mortality of CRC ranked second (12.2%) and fourth (9.5%) among all cancers in China. There is growing evidence that gut microbiota can alter the toxicity and effectiveness of CRC treatments [6–9].

Probiotics are live microorganisms that benefit the host when given in sufficient amounts [10]. Many probiotic genera are reported in CRC studies, including *Lactobacillus*, *Lactococcus*, *Bifidobacterium*, *Clostridium*, *Bacillus*, *Saccharomyces*, *Pediococcus*, and *Leuconostoc* [11–16]. The role of probiotics in CRC is becoming increasingly evident [12, 17–19]. By altering the composition of gut microorganisms, enhancing gut tight junctions, and changing the expression of intestinal inflammatory factors [13, 14, 20], probiotics improve intestinal function and reduce complications in CRC patients [9, 13, 15–18, 21–25]. However, since probiotics may cause sepsis, the safety of probiotics in cancer patients is also a continuing concern [26–29].

Clinical probiotic indications include diarrhea, constipation, indigestion, and gastroenteritis caused by gut microbiota dysbiosis [30, 31]. In 2011, an extensive telephone survey showed that 25.4% of 873 consumers in New Zealand used probiotics [32]. Most probiotic users (75.2%) took it on medical recommendations. Probiotics were primarily alongside antibiotic treatment (23%) and gastrointestinal disorders (27.5%). In a study of 145 US hospitals, probiotics were used in about 2.6% of hospitalizations in 2012 [33]. The top-used probiotic formulations were *Saccharomyces boulardii* (32%), *Lactobacillus acidophilus* and *Lactobacillus bulgaricus* (30%), *Lactobacillus acidophilus* (28%), and *Lactobacillus rhamnosus* (11%). Probiotic use increased significantly from 1.0% in 2006 to 2.9% in 2012. Another survey of probiotic use among patients at a tertiary medical center in the U.S. showed that 55% (333) responders had recently used probiotics, including food products (90% of probiotic users) and/or supplements (60%) [34, 35]. Most patients (56%) choose a probiotic mixture. Only a few patients (12%) were recommended for use by physicians. Another U.S. consumer survey conducted in 2017 found that 39.4% of responders (n = 396) used probiotics [36]. In 2017, a Slovakia prospective outpatient survey study reported for the first time the prevalence, side effects, and factors that most likely influence probiotic use in cancer patients [30]. In a database descriptive cross-sectional study involving 212 patients with and without cancer, probiotics were used by 9.9% of cancer patients and 7.3% of non-cancer patients before receiving integrative medicine and health consultations [37].

The usage of probiotics varies significantly from study to study due to the different subjects included and the different definitions of probiotic use (such as whether probiotic foods are included or not). The prevalence of probiotics usage in reality Chinese cancer patients, especially CRC patients, has not been reported. In addition, the most prescribed probiotic strains or mixtures in clinical research are not necessarily consistent with those in clinical practice. Hospital prescription sample research helps quantitatively estimate the current probiotic prescription trends in reality CRC patients. It provides the basis for clinical guidance, public health work, and research direction. Therefore, this study aimed to evaluate and characterize the hospital probiotic prescription trends in Chinses CRC patients.

## Materials and methods

### Study design and ethical approval

We extracted the prescription data retrospectively from the Hospital Prescription Analysis Cooperative Project (HPACP). The ethics committee of the Second Affiliated Hospital of Zhejiang University approved this study (2020–827) and the newly added prescription data from 2020 to 2021 as the revised content of the research plan (2023–167). The same committee waived informed consent as part of the approval.

### Data source and prescription information extraction

HPACP is a national multi-center hospital prescription database [38–46]. Each participating hospital randomly selects ten days from its prescription information and uploads it to this database every quarter. Different prescriptions for the same patient are defined as individual data. One hundred and one hospitals in eight major Chinese cities collected CRC prescriptions for this study. S1 Table describes the distribution and grade of hospitals.

Prescriptions that met the following criteria were included: (1) Prescriptions from hospitals located in eight major areas of China (Beijing, Chengdu, Guangzhou, Hangzhou, Shanghai, Shenyang, Tianjin, and Zhengzhou); (2) Prescriptions from secondary and tertiary hospitals that participated in the program continuously from 2015 to 2021; (3) Prescriptions written during 2015 and 2021; (4) Prescriptions written for patients with the diagnosis of colon, rectal, or colorectal cancer.

Data for 2015 to 2019 were collected from October 15, 2020, to October 20, 2020. Data for 2020 to 2021 were collected from February 22, 2023, to February 24, 2023. HPACP applied Microsoft Access for data storage, filtration and extraction. According to the inclusion criteria, there 1216700 prescriptions were initially included. After removing 295 prescriptions for patients under 18 and 96 prescriptions from first-class hospitals, 1216309 prescriptions for patients with colorectal cancer were left. Prescription code, patient gender, age, hospital location, diagnosis, inpatient or outpatient, generic name, and price of probiotics were collected. HPACP used prescription coding to de-identify patient information and protect patient identity. The authors could not identify individual participants during or after data collection. The ethics committees approved the prescription extraction of HPACP of participating hospitals. This study began on October 21, 2020.

### Statistical analysis

We applied Sankey Diagrams by RAWGraghs 2.0 (https://www.rawgraphs.io/) to visualize the source of prescriptions. We analyzed the consumption and expenditure of probiotics as a whole and separately according to different formulations and strains. The average cost per visit was the yearly probiotic expenditure divided by the number of probiotic prescriptions. The statistical significance of trends for the usage and cost of total probiotics was analyzed using the Mann-Kendall test. Other probiotic trends were estimated using the Cochran-Armitage trend test R V.3.3.0 (http://www.R-project.org). We demonstrate the mixture of different probiotic strains by Gephi software (version 0.9.4). Backward stepwise multivariable logistic regression with SPSS 26.0 for Windows (SPSS, Inc, Chicago, Ill) investigated risk factors for antitumor agents co-prescribed with probiotics. Variables with *p* < 0.2 in single-variable comparisons were included in a multivariable logistic regression model. A two-tailed *p* < 0.05 was considered to be statistically significant. Relative risk was estimated using odds ratios (ORs) with corresponding 95% confidence intervals (CIs). The Spearman’s rank correlation coefficient with SPSS 26.0 for Windows (SPSS, Inc, Chicago, Ill) calculated correlations between probiotic strain and antitumor drug usage. A *p*-value less than 0.05 is statistically significant.

## Results

### Overall trends and expenditure of probiotic prescriptions

There were 1216309 CRC prescriptions from 2415 departments of 101 hospitals. The number of CRC prescriptions varied significantly among the 101 hospitals, ranging from 0.0 to 378.5 per day, with an average of 43.0 ± 69.7 (S2 Table). The 15 hospitals with the most prescriptions were 190.9 prescriptions per day. In the remaining 86 hospitals, the average number of daily prescriptions was 17.2. Among them, 47290 prescriptions from 1089 departments of 93 hospitals contained probiotics.

According to co-prescription drugs and diagnostic analysis, 48.3% of probiotic prescriptions were without gastrointestinal symptoms (OR 0.57, 0.56–0.58, *p* < 0.001). Besides, gastroenteritis (27.7%, 1.38, 95% CI 1.35–1.41, p < 0.001) was the most common gastrointestinal symptom for probiotic prescriptions, followed by constipation (13.5%, 1.34, 1.30–1.38, *p* < 0.001), diarrhea (13.4%, 10.91, 10.57–11.26, *p* < 0.001), gastrointestinal dysfunction (12.5%, 0.77,0.75–0.79, p < 0.001), dyspepsia (8.8%, 3.97, 3.82–4.12, *p* < 0.001). In addition, tertiary hospital (1.43,1.36–1.50, *p* < 0.001), outpatient (1.30, 1.28–1.33, *p* < 0.001), older CRC patients (1.018 per year, 1.017–1.019, *p* < 0.001) prescriptions were more likely to have probiotics.

There were 21 probiotic products identified, including 13 formulations in 20 dosage units and 15 probiotic strains (S3 Table). All products are Chinese Pharmacopoeia listed. The probiotic strains come from healthy human flora or other species [47]. Over the seven years, the annual probiotic prescription number increased by 82.1%, and the proportion of probiotic prescriptions increased significantly from 3.3% to 4.2% (Z = 12.771, *p* < 0.001, Table 1). Meanwhile, the yearly and average visit cost of probiotics increased by 109.0% (z = 3.00, *p* = 0.003) and 14.8% (z = 2.40, *p* = 0.016), respectively.

**Table 1:** The overall trend of probiotic prescriptions for colorectal patients in China, 2015–2021.

Year	2015	2016	2017	2018	2019	2020	2021	*p* for trend
**All prescriptions, n**	144815	155406	179122	177654	174704	180099	204509	0.036
**Probiotic prescriptions, n(%)** ^**a**^	4767(3.3)	5307(3.4)	6385(3.6)	7182(4.0)	7607(4.4)	7362(4.1)	8680(4.2)	0.016
**Total probiotic prescription expenditure, CNY** ^**b**^	282117.5	298178.1	375234.6	451295.7	485489.3	488398.4	589585.1	0.003
**Probiotic cost per prescription, CNY** ^**b**^	59.2	56.2	58.8	62.8	63.8	66.3	67.9	0.016
**Probiotics co-prescribed with antitumor drugs, n(%)** ^**c**^	315(6.6)	385(7.3)	592(9.3)	867(12.1)	978(12.9)	1023(13.9)	1195(13.77)	<0.001
**Probiotics co-prescribed with antibiotics, n(%)** ^**c**^	656(13.8)	804(15.1)	1011(15.8)	949(13.2)	1120(14.7)	1012(13.8)	1252((14.4)	<0.001
**Probiotics co-prescribed with antitumor drugs and antibiotics, n(%)** ^**c**^	25(0.5)	45(0.9)	38(0.6)	59(0.8)	57(0.8)	41(0.6)	57(0.7)	0.368

^a^The denominator is the number of all prescriptions in the year.

^b^CNY, Chinese Yuan.

^c^The denominator is the number of probiotic prescriptions in the year.

In 2015, there was 6.6% of probiotics co-prescribed with antitumor drugs. In 2021, it increased to 13.8% (Z = 15.31, *p* < 0.001). At the same time, the proportion of probiotics co-prescribed with antibiotics or antibiotics plus antitumor drugs was relatively stable. Probiotics co-prescribed with antibiotics accounted for about 15% of all prescriptions (Z = 4.34, *p* < 0.001). Less than 1% of probiotics were co-prescribed antitumor drugs and antibiotics (Z = 0.90, *p* = 0.368).

### Descriptive statistics of probiotic prescriptions

Table 2 shows the demographic data of the probiotic prescriptions. Male patients’ prescriptions accounted for 61.5% of the seven-year probiotic prescriptions. The probiotic prescriptions for patients under 40 was the least (3.5%), followed by patient aged 40–49 (7.8%) within seven years. Gender and age distribution were relatively stable during the study period. The mean patient age of probiotic prescriptions was 65.9 ± 13.7 years over seven years. Among them, patients co-prescribed antitumor drugs (60.3 ± 11.8, *p* < 0.001) or antibiotics (63.8 ± 12.8, *p* < 0.001) were younger. There was no significant difference in gender.

**Table 2:** Descriptive statistics of probiotics prescriptions for colorectal cancer in China, 2015–2021^a^.

Year		2015	2016	2017	2018	2019	2020	2021
**Gender**	Male	60.9	61.1	60.8	61.3	60.8	61.8	62.9
	Female	39.1	38.9	39.2	38.7	39.2	38.3	37.1
**Age**	0–39	2.9	3.3	4.0	3.7	3.3	3.4	3.6
	40–49	9.3	8.4	7.4	7.6	8.1	7.8	6.7
	50–59	18.1	19.2	18.3	19.1	19.7	20.6	20.0
	60–69	30.3	28.7	29.6	32.3	31.5	31.1	30.5
	70–79	20.9	22.2	21.7	21.5	22.9	22.8	21.5
	80-	18.5	18.2	19.0	15.9	14.4	14.2	17.7
**Cancer diagnosis**	Colon cancer	51.2	52.7	52. 6	51.1	50.0	50.8	52.5
	Rectal cancer	48.3	46.8	46.8	48.5	49.6	48.0	46.3
	Site unspecified	0.5	0.5	0.6	0.4	0.4	1.1	1.2

^a^Statistics are shown as percentage unless otherwise noted.

**Fig 1:**
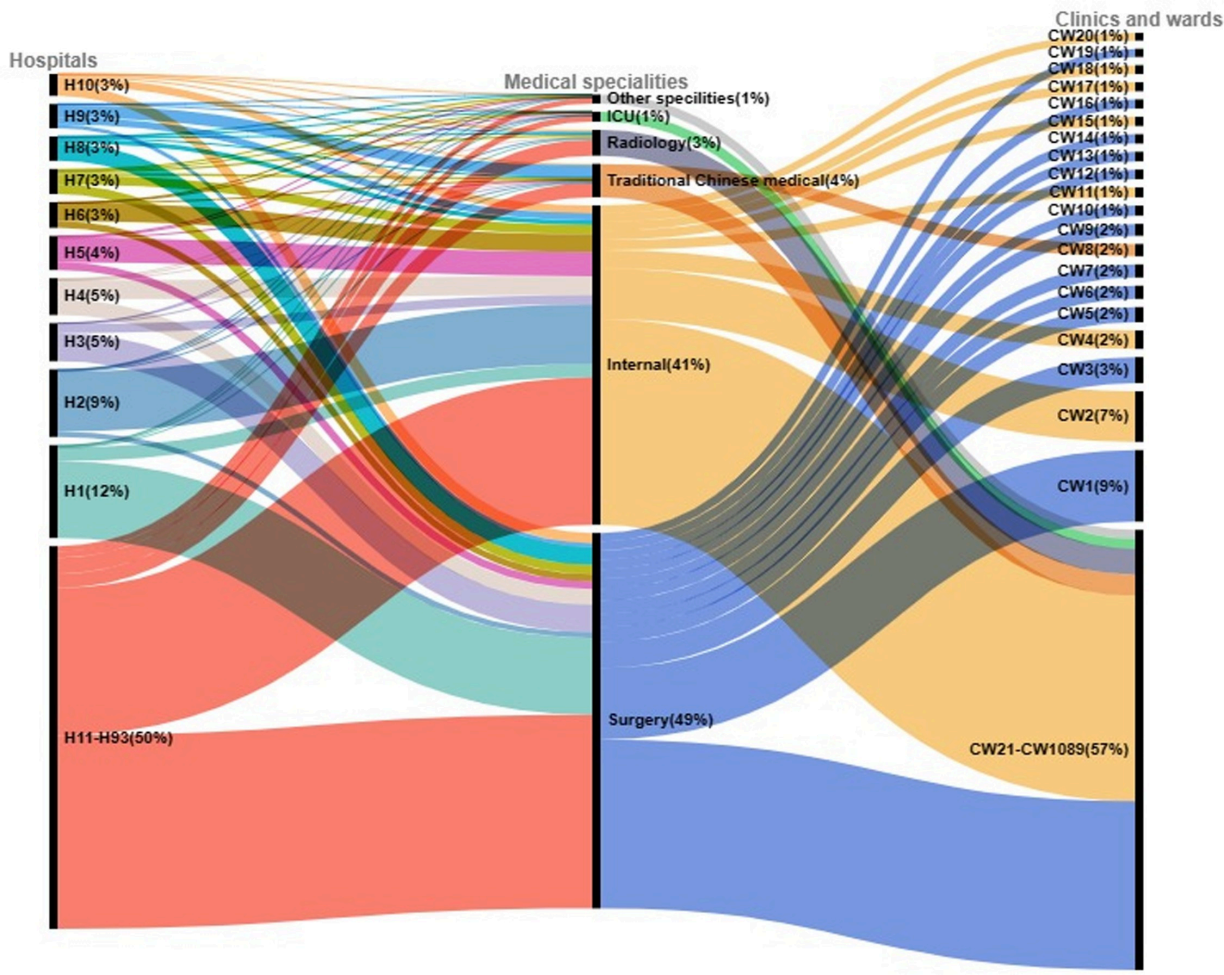
The Sankey diagram depicts the hospital, specialty, and medical department of probiotic prescriptions. Hospitals and medical departments are listed in descending order according to the probiotic prescription number. H1 is the hospital with the most probiotic prescriptions. CW1 is the department with the most probiotic prescriptions. Percentages in parentheses are the percentage of total probiotic prescriptions over seven years.

As shown in Fig 1, probiotic prescriptions varied considerably across hospitals and medical departments. The proportion of probiotic prescriptions in different hospitals ranged from 0 to 12 percent. The top 10 hospitals and top 20 departments accounted for 50.5% and 43.0% of probiotic prescriptions. In addition, the number of probiotic prescriptions varied by medical specialty. Surgery accounted for 48.6%, and internal medicine for 41.3%. Radiology, traditional Chinese medicine, intensive critical care, and other specialties accounted for 10.1% of the total.

### The prescribing trend of probiotic formulations

**Fig 2:**
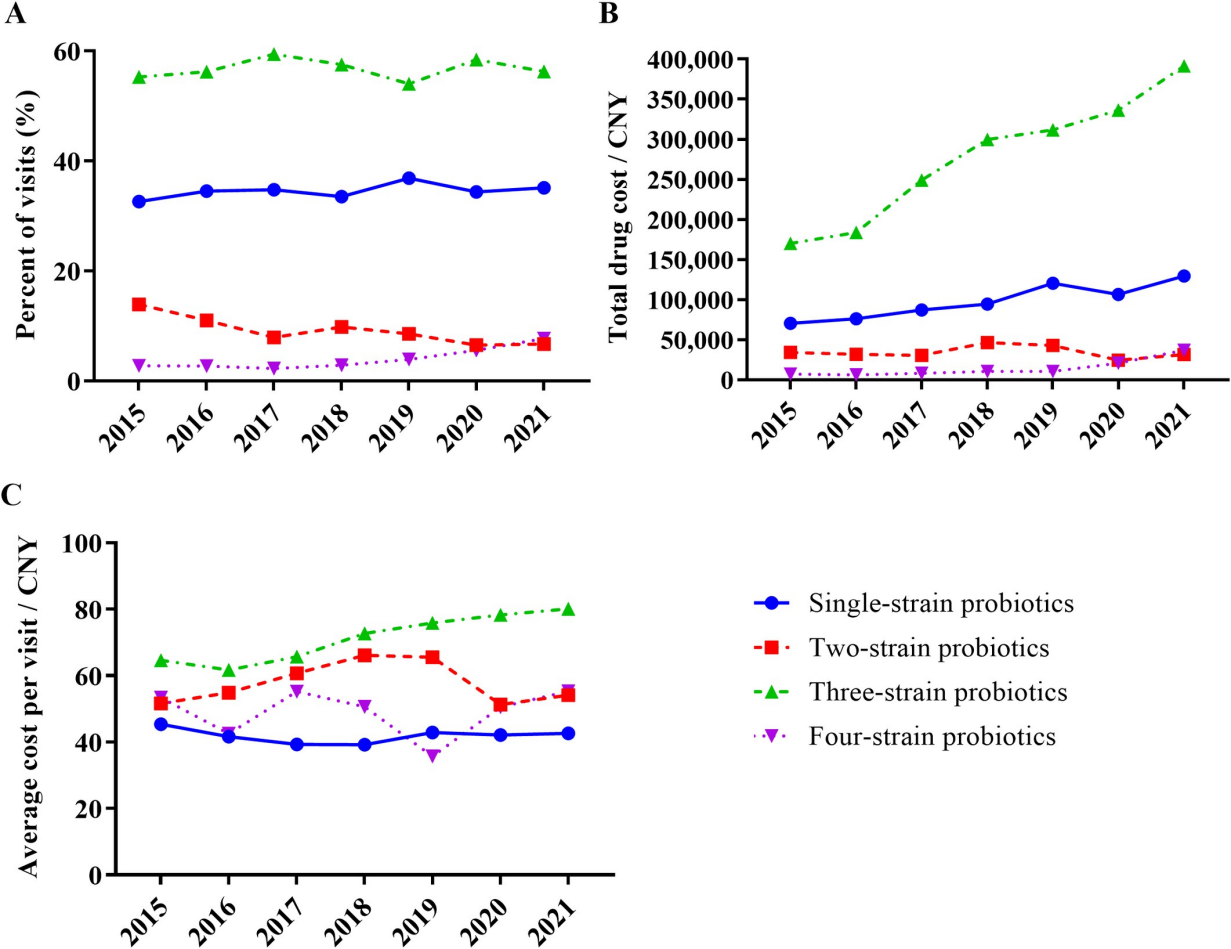
Overall prescribing trends of probiotic formulations in Chinese colorectal patients. (A) The proportion of probiotic prescriptions; (B) The total probiotic prescription cost; (C) The average cost per probiotic prescription.

According to different strain numbers of probiotic formulations (S3 Table), prescriptions were classified and analyzed. Three-strain probiotic formulations ranked first in percent of visits (PV), as shown in Fig 2A, accounting for 56.7% of probiotic prescriptions during the study period (Z = 0.03, *p* = 0.978). The PV of single-strain probiotics was 34.7% (Z = 1.21, *p* = 0.225). Two-strain probiotics were the only class of probiotics in which PV decreased from 13.9% in 2015 to 6.7% in 2021 (Z = -9.87, *p* < 0.001). Four-strain probiotic PV increased from 2.8% in 2015 to 7.7% in 2021 (Z = 11.36, *p* < 0.001).

The ranking of total probiotic expenditure was consistent with the order of prescription quantity. From 2015–2021, ¥1.94 million (Chinese currency Yuan) was spent on three-strain probiotics, making it the highest class in the total expenses (Fig 2B) and per capita expenses (Fig 2C). Single-strain probiotics ranked second in all four classes, costing ¥ 0.69 million. Two-strain and four-strain probiotic products cost 0.24 and 0.10, respectively. The yearly expense for three-strain (Z = 19.82, *p* < 0.001) and four-strain (Z = 75.33, *p* < 0.001) probiotic products increased significantly during 2015–2021. Meanwhile, the total yearly expense of single-strain (Z = -81.66, *p* < 0.001) and two-strain (Z = -81.55, *p* < 0.001) probiotic products dramatically declined.

**Fig 3:**
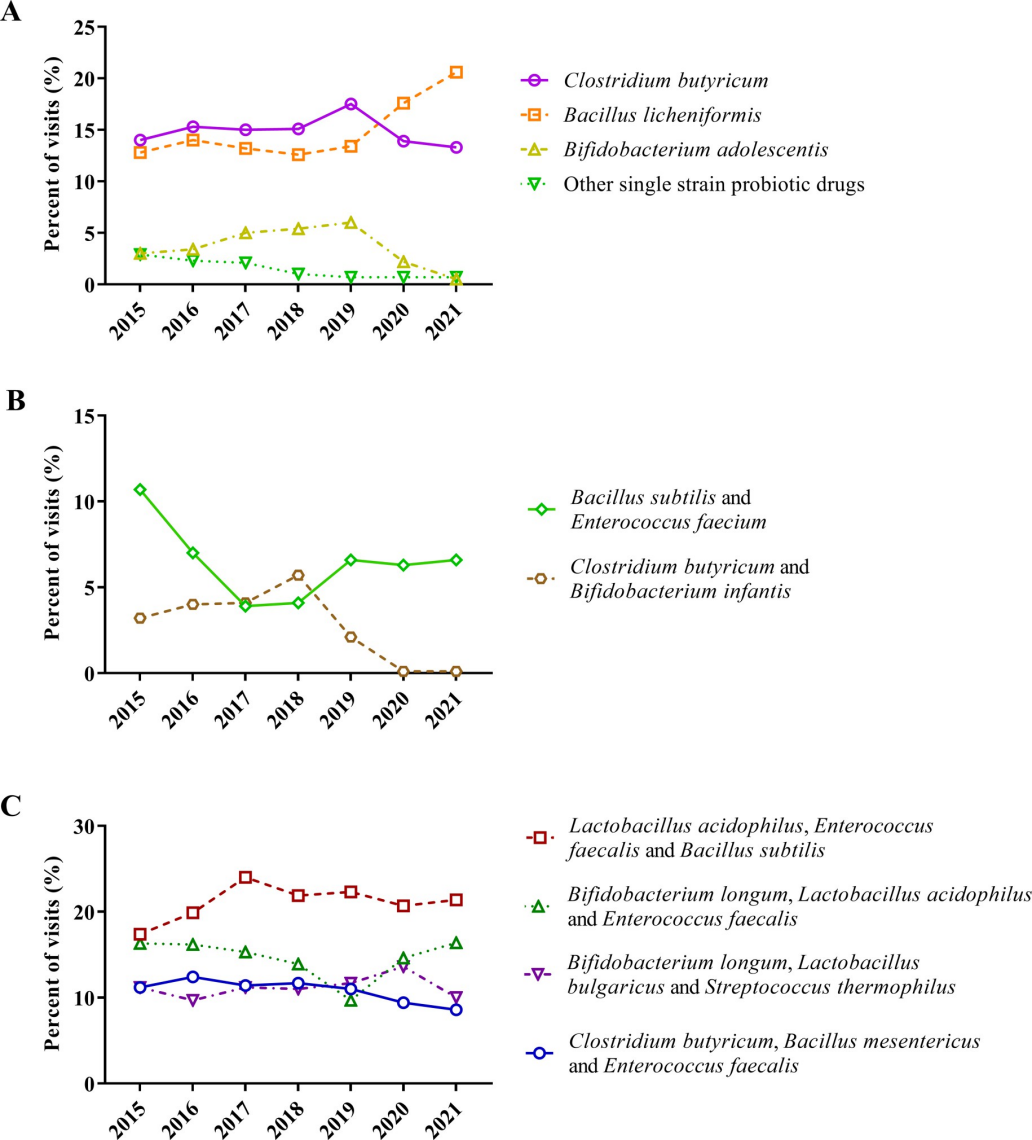
Individual prescribing trends of probiotic formulations in Chinese colorectal patients. (A) Single-strain formulations; (B) Two-strain formulations; (C) Three-strain formulations.

The prescribing trends of individual probiotic formulations were analyzed. There were a total of six single-strain probiotic formulations identified. *Bacillus licheniformis* (15.2%, Z = 8.08, *p* < 0.001), *Clostridium butyricum* (14.9%, Z = -1.09, *p* = 0.277), and *Bifidobacterium adolescentis* (3.6%, Z = -6.53, *p* < 0.001) (Fig 3) were the most prescribed single-strain probiotic fornulations, The PV of *Bacillus coagulans* (0.4%, Z = -10.03, *p* < 0.001), *Bacillus Cereus*, or *Saccharomyces Boulardii* was less than 0.5% (0.5%, Z = 0.01, *p* = 0.99), with a non-increasing prescribing trend.

Within the study years, there was a decrease in the use of two-strain probiotic formulations. *Bacillus subtilis* and *Enterococcus faecium* accounted from 10.7% to 6.6% (Z = -2.65, *p* = 0.008). *Clostridium butyricum* and *Bifidobacterium infantis* declined from 3.2% to 0.1% (Z = -13.08, *p* = 0.008).

The number of prescriptions for the three-strain formulas remained stable. *Lactobacillus acidophilus*, *Streptococcus faecalis*, and *Bacillus subtilis* were the most prescribed probiotic mixture in this study, which accounted for 21.3% PV in total (Z = -1.65, *p* = 0.100). *Bifidobacterium longum*, *Lactobacillus acidophilus*, and *Enterococcus faecalis* accounted for 14.3% (Z = -1.32, *p* = 0.187) of probiotic prescriptions. The seven-year PV of *Bifidobacterium longum*, *Lactobacillus bulgaricus*, and *Streptococcus thermophilus* was 11.2% (Z = 1.02, *p* = 0.307). The seven-year PV of *Clostridium butyricum*, *Bacillus mesentericus*, and *Enterococcus faecalis* was 10.9% with a decreasing trend (Z = -4.40, *p* < 0.001).

The only four-strain probiotic formulation consisted of *Bifidobacterium infantis*, *Lactobacillus acidophilus*, *Enterococcus faecalis*, and *Bacillus cereus*. The PV of this formulation significantly increased from 2.8% to 7.7% (Z = 11.36, *p* < 0.001).

### Trends of probiotic prescribing patterns

Most prescriptions contained just a single probiotic product (95.0%, Z = 0.124, *p* = 0.902, S4 Table) but multi strains (69.3%, Z = -0.36, *p* = 0.719). The combined use of probiotic strains was consistent with the trend of formulation application. There were 30.7% single-strain probiotic prescriptions (Z = -0.615, *p* = 0.538). Three-stain combination accounted for 52.5% of probiotic prescriptions (Z = 0.436, *p* = 0.663). The two-strain combination decreased from 13.4% to 6.1% (Z = -9.49, *p* < 0.001). Prescriptions containing four or more probiotic strains increased from 6.2% to 12.0% (Z = 6.28, *p* < 0.001).

There were the prescribing trends of specific probiotic strains in the S4 Table. *Enterococcus spp*, *Bacillus spp*, *Lactobacillus spp*, and *Bifidobacterium spp* were the most prescribed probiotic genus. *Enterococcus faecalis*, *Lactobacillus acidophilus*, *Bacillus subtilis*, *Bifidobacterium longum*, and *Clostridium butyricum* were the most prescribed probiotic strains, accounting for 50.6% (Z = 1.36, *p* = 0.173), 40.0% (Z = 3.59, *p* < 0.001), 27.6% (Z = 0.25, *p* = 0.799), 25.7% (Z = -0.313, *p* = 0.754), and 17.4% (Z = -5.70, *p* < 0.001), respectively. Among them, the prescription of *Bacillus cereus* (4.7%, Z = 8.85, *p* < 0.001), *Bifidobacterium adolescentic* (5.1%, Z = 5.16, *p* < 0.001), and *Lactobacillus acidophilus* increased over seven years. Other probiotic strain usage remained stable or decreasing trend.

**Fig 4:**
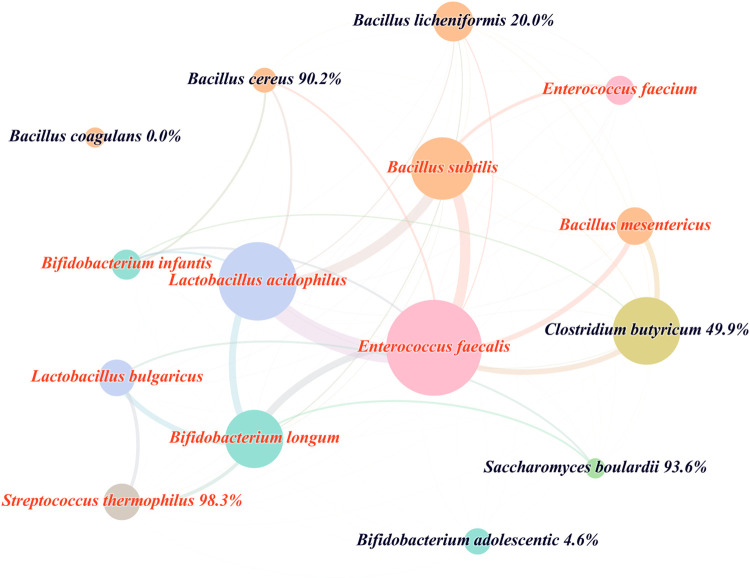
Node-edge diagram of probiotic strains prescribed in combination. Node labels consist of the probiotic strain name and the percentage of this strain co-prescribed with other strains. Node color indicates probiotic genus. Node size corresponds to the prescription number of strains. The red node label color indicates that the probiotic strain is 100% co-prescribed with other strains, while black means, not 100%. The thickness of the edges is the number of co-prescriptions of probiotic strains at both ends.

To identify the co-prescribing of different probiotic strains, we applied Gephi software for network analysis. As shown in Fig 4, nine of the 15 probiotic strains were never used alone (red node label) because there are no single-strain formulations for these nine strains. The most prescribed of these nine strains is *Enterococcus faecalis*, which consists of four formulations, including the most prescribed formulation. The remaining six strains were prescribed alone or in combination (black node label). *Bacillus coagulans* and *Clostridium butyricum* form multi-strain formulations with other probiotics. Therefore, there was a higher co-prescribed rate of *Bacillus cereus* (90.2%) and *Clostridium butyricum* (49.9%) than *Bacillus licheniformis* (20.0%), *Bifidobacterium adolescentis* (4.6%) and *Bacillus coagulans* (0.0%). *Saccharomyces boulardii* is the only fungus probiotic in this study, which also had a high proportion of combination (93.6%) with other probiotic strains.

### Prevalence and influence factors of probiotics co-prescribed with antitumor drugs

Over the seven years, the proportion of probiotic prescriptions co-prescribed with antitumor drugs increased gradually from 6.6% to 13.8% (Z = 15.31, *p* <0.001, Table 1). To further clarify the reasons for the increase in co-prescription, we collected 189030 antitumor drug prescriptions without probiotics prescriptions for analysis. As shown in Table 3, the proportion of specific antitumor drugs co-prescribed with probiotics varies. Doxifluridine had the highest co-prescription rate of 8.7%, and tegafur had the lowest co-prescription rate of 0.4%.

**Table 3:** Number of antitumor drug prescriptions co-prescribed with probiotics for Chinese colorectal patients.

	With probiotics, n (%)	Without probiotics, n (%)
**Doxifluridine**	220(8.7)	2305(91.3)
**Capecitabine**	3655(3.8)	92409(96.2)
**Tegafur, gimeracil and oteracil potassium (TGO)**	465(3.7)	12011(96.3)
**Regorafenib**	52(3.0)	1658(97.0)
**Bevacizumab**	493(2.9)	16493(97.1)
**Irinotecan**	557(2.5)	21512(97.5)
**Oxaliplatin**	1114(2.1)	52116(97.9)
**Cetuximab**	147(1.9)	7756(98.1)
**Fluorouracil**	634(1.7)	37092(98.3)
**Raltitrexed**	62(1.3)	4565(98.7)
**Tegafur**	9(0.4)	2199(99.6)

**Fig 5:**
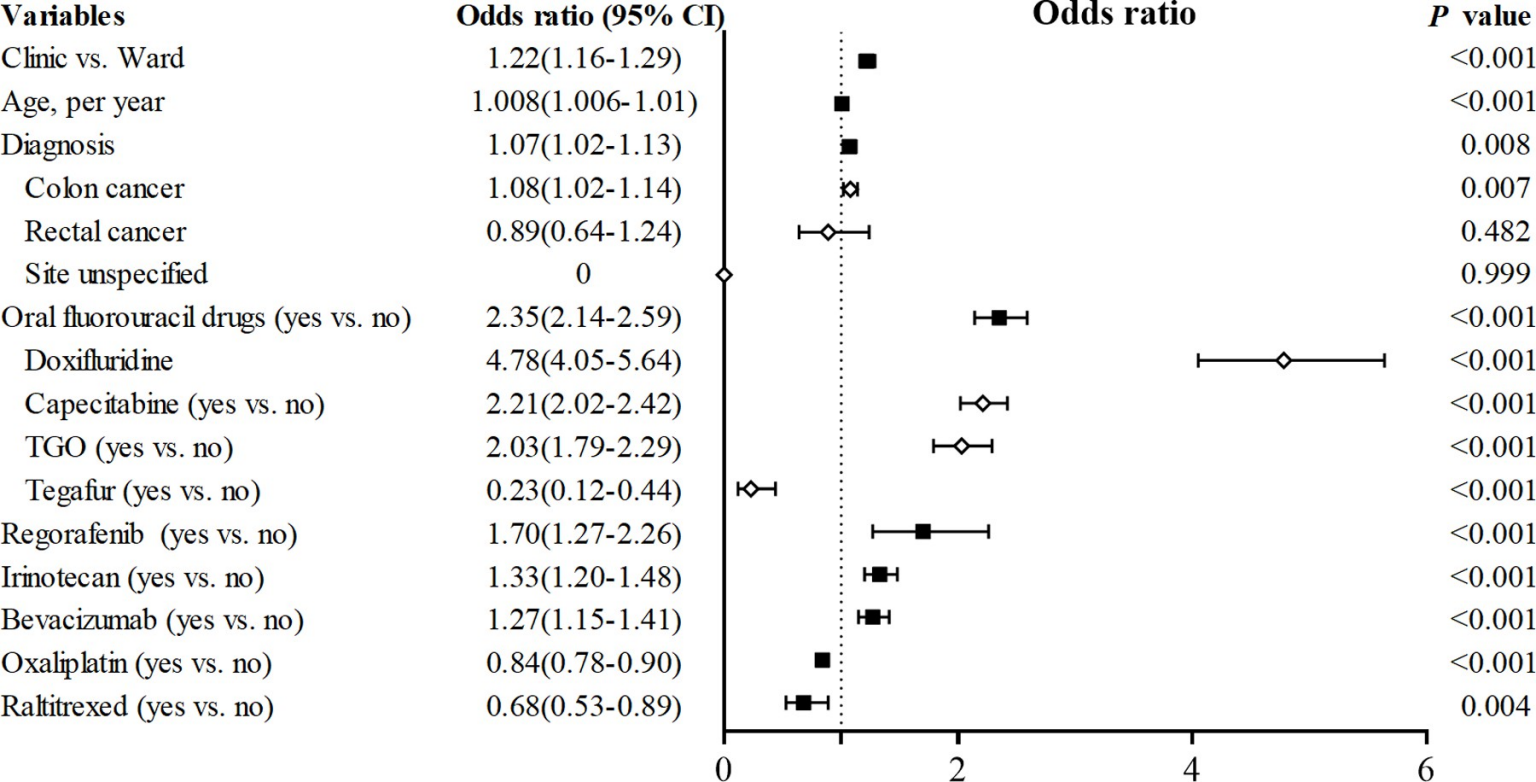
Forest plot comparing the risk factors of antitumor drug co-prescribed with probiotics. Odds ratio on the X-axis, values <1 confer antitumor drug prescribed without probiotic, values >1 confer antitumor drug co-prescribed with probiotic. The confidence interval is a horizontal black line for each variable. The black box or the white rhombus represents the odds ratio referenced. TGO, tegafur, gimeracil and oteracil potassium.

To identify risk factors for co-prescribing antineoplastic drugs and probiotics. Binary regression analysis included the age, gender, diagnosis, source of prescription (clinic or ward), hospital grade, and co-prescribed antineoplastic drugs. There were 236320 prescriptions in the analysis. The main risk factors identified for co-prescribing were the source of prescription, patient age, diagnosis, and specific antitumor drug (Fig 5). Outpatients (OR 1.22, 95% CI 1.16–1.29, *p* < 0.001), senior patients (1.008 per year, 1.006–1.011, *p* < 0.001), and colon cancer patients (1.08, 1.02–1.14, *p* = 0.007) were more likely to receive probiotics along with antitumor therapy. The specific antitumor drug used mattered more. Doxifluridine (4.78, 4.05–5.64, *p* < 0.001), capecitabine (2.21, 2.02–2.42, *p* < 0.001), TGO (2.03, 1.79–2.29, *p* < 0.001), regorafenib (1.70, 1.27–2.26, *p* < 0.001), irinotecan (1.33, 1.20–1.48, *p* < 0.001), and bevacizumab (1.27, 1.15–1.41, *p* < 0.001) were more likely to prescribe with probiotics.

**Fig 6:**
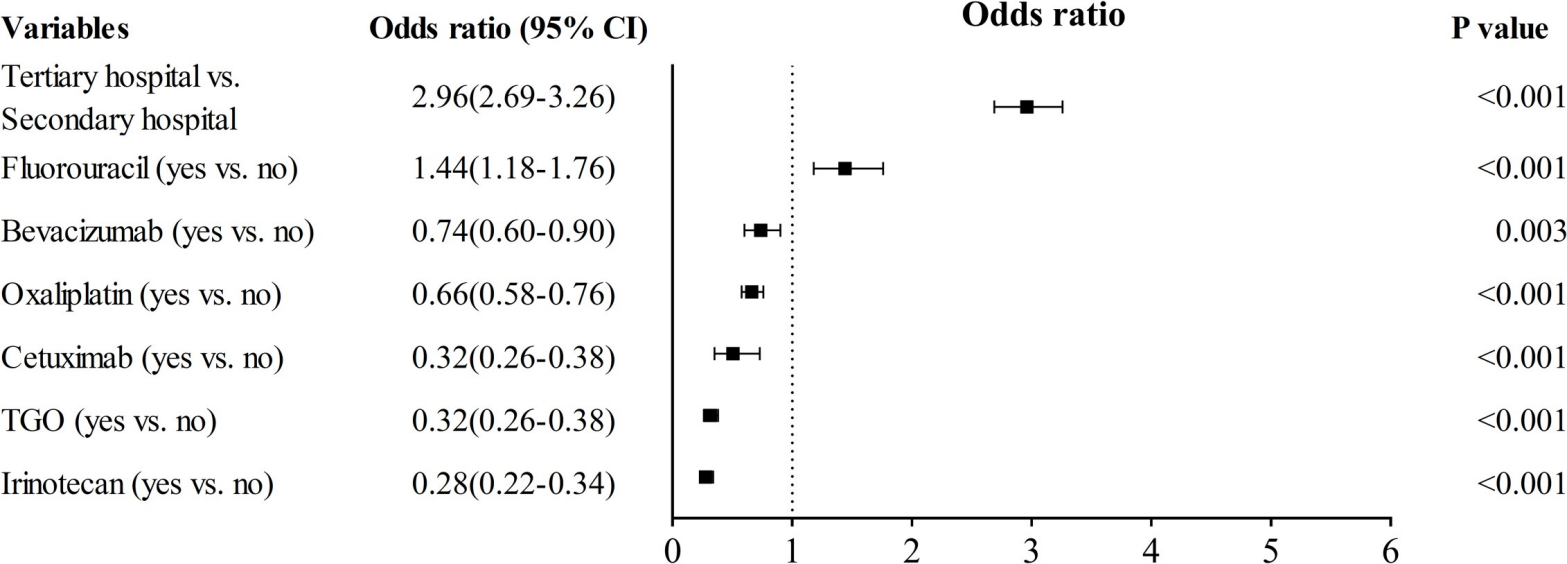
Forest plot comparing the risk factors of antitumor drug co-prescribed with multi-strain probiotics. Odds ratio on the X-axis, values <1 confer antitumor drug co-prescribed with single strain probiotic, values >1 confer antitumor drug co-prescribed with the multi-strain probiotic. The confidence interval is a horizontal black line for each variable. The black box represented the odds ratio referenced to the X-axis. CI, confidence interval. TGO, tegafur, gimeracil and oteracil potassium.

To identify the factors influencing the selection of single or multiple strains of probiotics co-prescribed with antitumor drugs, we conducted a binary linear regression analysis of 47290 probiotic prescriptions. The binary regression analysis included the age, gender, diagnosis, source of prescription (clinic or ward), hospital grade, and co-prescribed antitumor drugs. The prescriptions with multi-strain probiotics were more likely from tertiary hospitals (2.96, 2.70–3.26, *p* < 0.001) (Fig 6) or co-prescribed with fluorouracil (1.44, 0.22–0.34, p < 0.001). Meanwhile, irinotecan (0.28, 0.22–0.34, *p* < 0.001), TGO (0.32, 0.26–0.38, p < 0.001), cetuximab (0.51, 0.35–0.73, *p* < 0.001), oxaliplatin (0.66, 0.58–0.76, *p* < 0.001), and bevacizumab (0.74, 0.60–0.90, *p* = 0.003) were more like to co-prescribed with single-strain probiotics.

**Fig 7:**
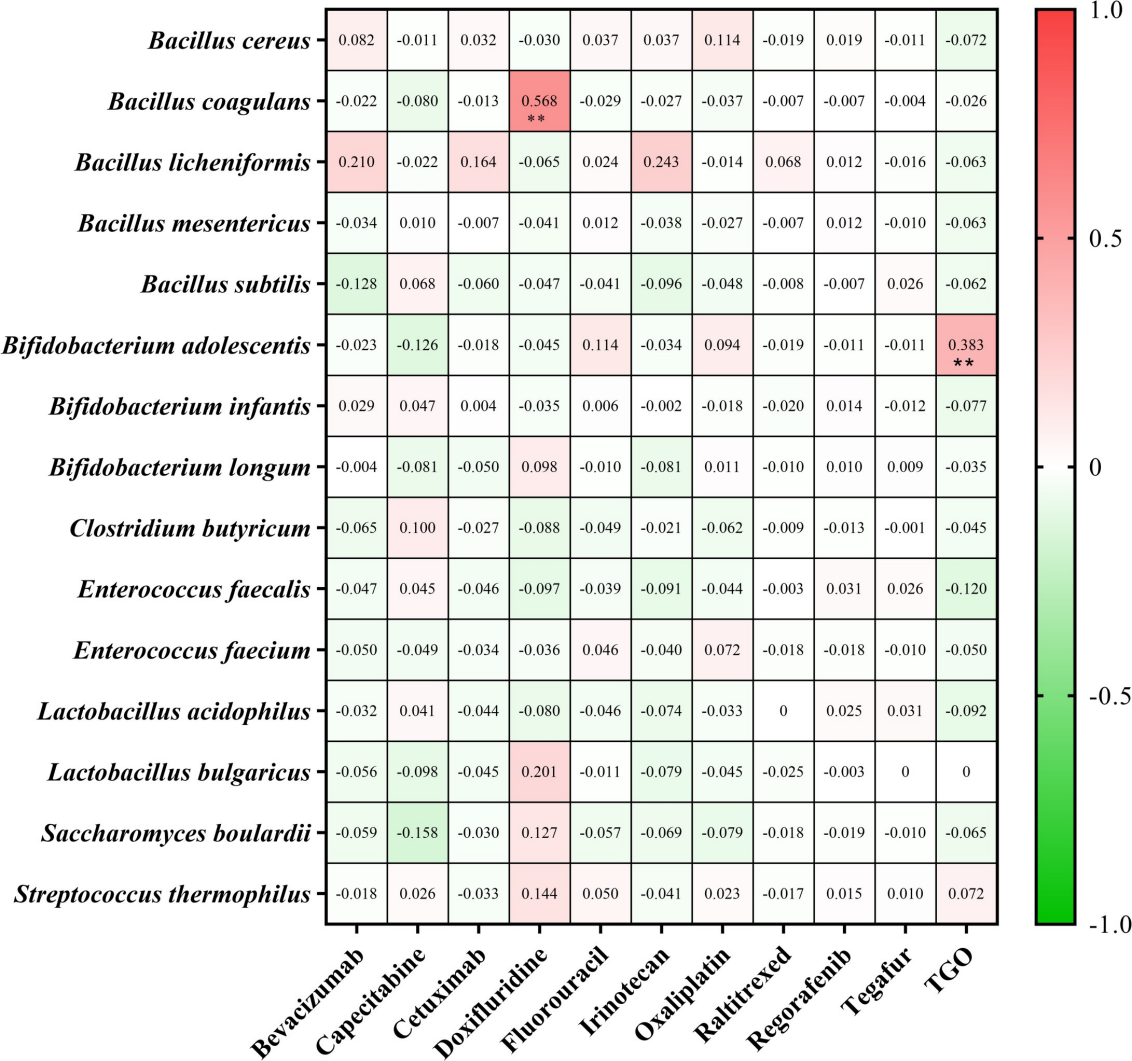
Heat map of correlation coefficients between probiotic strain and antitumor drugs. The probiotic strains are in the rows, and the antitumor drugs are in the columns. The grid color represents a positive (red) or negative (green) correlation. The closer the absolute value of the correlation coefficient to 1, the higher the color saturation. The numbers in the grid are the correlation coefficients between probiotics and antitumor drugs on the corresponding vertical axis. **, *p* < 0.001. The *p*-value is not marked unless the absolute value of the correlation coefficient is more than 0.3. TGO, tegafur, gimeracil and oteracil potassium.

Finally, we explored the correlation between specific antitumor drugs and probiotic prescribing. We included 5681 antitumor drugs and probiotic co-prescribed prescriptions for correlation analysis. We applied Spearman’s rank correlation to assess the association between anticancer drugs and probiotic strains. Take a correlation coefficient greater than 0.8 as a strong correlation. There was no strong correlation (correlation coefficient ≥ 0.8) found between particular probiotics and antitumor drugs (Fig 7).

## Discussion

In this study, which included 101 hospitals across China, a fair amount of CRC prescriptions contained probiotics. According to 2019 data from the Institute for Health Metrics and Evaluation-Global Burden of Disease (IHME-GBD), there are over 3.5 million CRC patients in China, accounting for 31% of the global total. The proportion of probiotic prescriptions was not high in this study. However, considering the total number of CRC patients in China, many patients might have received probiotics in medical services. Moreover, probiotics used in Chinese CRC patients showed a significant upward trend, similar to those previously reported in inpatients in the United States [48].

Between 2015 and 2021, probiotics accounted for 3.3% to 4.4% of Chinese CRC prescriptions. A previous survey reported that the probiotic utilization rate among cancer outpatients in Slovakia was 28.5% [30]. Since we only included hospital prescriptions, the Slovakia study included probiotic foods and dietary supplements, which may be reasonable for our study’s lower rate of probiotics. Another 2012 tally of discharged patients in the U.S. showed that 2.6% of children and adult patients had used probiotics during their hospital stay. The hospital’s proportion of discharged patients treated with probiotics varied from 0.3% to 8.5% [48]. In contrast to the U.S. study (non-cancer patients), our study included prescriptions for CRC patients in which gastrointestinal disorders are common. The main indication for probiotics is gastrointestinal disorders [49]. So, it may be reasonable that CRC patients have a higher proportion of probiotic prescriptions. Therefore, the difference in probiotic prevalence might be acceptable in our study population.

The prescriptions of the most prescribed seven probiotic formulations came from 98.7% of hospitals in our study. This result is comparable with the previously mentioned U.S. study [48], in which seven probiotic formulations came from 96% of the 145 hospitals. Meanwhile, each hospital in our study prescribed an average of 3.3 probiotic formulations (3.3±1.4; range 1–7) in 2021, higher than the reported U.S. hospitals, which may be related to the relatively large number (n = 15) of probiotic formulations in the Chinese pharmacopeia.

At the strain level, U.S. probiotics are significantly different from Chinese probiotics. Previous research has reported that the five most commonly used probiotics in U.S. inpatients were *Lactobacillus rhamnosus*, *Lactobacillus gasseri*, *Lactobacillus bulgaricus*, *Lactobacillus acidophilus*, and *Saccharomyces boulardii* [48, 50]. In CRC randomized controlled trials (RCTs), *Bifidobacteria* and *Lactobacillus* were the most popular probiotics [18]. The most prescribed strains in our study were *Enterococcus faecalis*, *Lactobacillus acidophilus*, *Clostridium butyricum*, *Bacillus subtilis*, and *Bifidobacterium longum*. Although the probiotic genera in our study were similar to the U.S. study, the probiotic strains were different. Due to the influence of different regions [51], diets, human genotypes, and listed probiotic strains, these differences may be acceptable.

To our knowledge, no guidelines recommend probiotics in CRC patients. However, many clinical studies have explored the effectiveness of probiotic therapy in CRC patients [16, 21, 22, 52].

There needs to be more research evidence for specific probiotic strains listed in China. *Lactobacillus acidophilus* and *Bifidobacterium longum* mixture could reduce cancer treatment-related mucositis [14]. As a widely used probiotic in multi-strain probiotic formulations in China, the role of *Enterococcus spp*. in CRC is still controversial [53, 54]. Preclinical studies have shown positive results regarding *Clostridium butyricum* and *Bacillus subtilis* in animal CRC models [55–58]. However, clinical evidence for using these probiotics in CRC patients still lacks [59].

Some studies showed that probiotic mixtures might provide more biological activities and benefits [21, 60]. Perhaps because of such considerations, about 70% of probiotic prescriptions were multi-strain in our study. However, other studies believe that multiple strains do not bring more benefits [61]. It may explain the other 30% single strain prescriptions. The decrease in two-strain probiotic prescriptions may be related to the lack of recommendations for such formulations (*Enterococcus faecium* and *Bacillus subtilis*). I Neither the 2016 Chinese consensus clinical application of microecological modulators for the digestive tract (in Chinese) nor the 2017 Chinese evidence-based guidelines for pediatric clinical application of probiotics (in Chinese) recommended this formula. There is also consensus on recommendations for specific probiotic formulation (live combined *Bifidobacterium*, *Lactobacillus* and *Enterococcus*) for use in cancer patients [62].

Since most prescriptions contained one probiotic product in our study, the prescribing pattern of probiotic strains is highly related to the formulation of multi-strain products. Although there is some evidence of multi-strain formulations in CRC patients [18, 21], the multi-strain formulations in our study were significantly different from those in previous studies. Overall, evidence for using these probiotics in cancer, particularly colorectal cancer, remains limited. Therefore, more research is needed to explore the role of these multi-strain formulations in CRC patients.

The relationship between probiotics and antitumor drugs is attracting increasing attention [19, 63]. We estimated the reality connection between antitumor agents and probiotic prescribing. Overall, antitumor drugs with higher gastrointestinal toxicities were more likely to be co-prescribed with probiotics. It may be related to the higher incidence of mucositis and diarrhea of fluorouracil drugs [64]. However, there was no increase in co-prescribing probiotics for fluorouracil injection in our study. It may be related to the much longer duration of administration and the higher diarrhea incidence of oral fluoropyrimidines [64, 65]. The number of prescriptions for tegafur, another oral fluorouracil, declined significantly over seven years, perhaps contributing to the low co-prescribing probiotic risk. Diarrhea occurred in 34% of patients treated with regorafenib, with 7% experiencing grade 3 or 4 [66]. Irinotecan also has high gastrointestinal toxicity [67]. Bevacizumab rarely causes diarrhea and mucositis, but it may be co-prescribed with other gastrointestinal toxic agents.

The mixture of probiotic strains might be more efficacious [21]. We analyzed the influencing factors of the multi-strain combination when co-prescribed with antitumor drugs. The results showed that prescriptions from tertiary hospitals were the most prominent driving factor for multi-strain probiotic use. It may be related to the rapid updating of doctors’ treatment concepts in tertiary hospitals.

Although some studies reported that changes in specific gut microbiota in CRC treatment are associated with treatment toxicity [68]. Our analysis found no association between specific antitumor agents and probiotic strains prescribing. It may be related to the unavailability of probiotic strains used in many studies and the lack of guidelines for the clinical use of local probiotic products in China cancer patients, which makes it difficult for physicians to select probiotic strains based on sufficient research evidence.

Our study described the current status and trends of probiotic prescriptions and expenditure of Chinese CRC patients for the first time. There are limitations to this study. First, due to the prevalence of prescription omissions in clinical indications, we indirectly obtained the possible reasons for using probiotics by analyzing co-prescribed drugs for other gastrointestinal symptoms, which may lead to some bias in the results. Second, since hospitals with HPACP locate in large cities, sampling might be biased. Third, we evaluated the probiotic use based on hospital data, the prevalence represented only physician prescriptions of probiotics.

The increase in probiotic prescriptions in Chinese CRC patients may suggest that more doctors believe in the benefits of probiotics. However, insufficient clinical evidence supports the use of currently available probiotics in such medical settings [69]. Follow-up studies should pay more attention to the efficacy, safety, dosage, timing, and course of treatment of specific probiotics for CRC patients.

In conclusion, the present study shows that probiotic prescription is increasing in Chinese colorectal patients. The increasing trends may be related to co-prescription growth with anticancer drugs. With unapproved indications and a lack of strain selectivity, evidence-based guidelines are urgently needed to improve probiotic use in this population.

## Supporting information

S1 Table Distribution and grade of hospitals (n = 101). (DOCX)

S2 Table Number of prescriptions from different hospitals (n = 101). (DOCX)

S3 Table Formulations of probiotic. (DOCX)

S4 Table Application trend of probiotics in prescribing patterns for colorectal cancer patients. (DOCX)

S1 Checklist STROBE statement—checklist of items that should be included in reports of observational studies. (DOCX)
