# Supplementary material for: Prevalence and influencing factors of probiotic usage among colorectal cancer patients in China: A national database study
Source: PLoS One. 2023 Sep 21;18(9):e0291864. doi: 10.1371/journal.pone.0291864 (PMC10513277; doi:10.1371/journal.pone.0291864)
Supplement: S1 Table — (DOCX) [file pone.0291864.s001.docx]

**Supplementary table 1. Distribution and grade of hospitals (n= 101)**

| **Location** | **Secondary Hospital (n)** | **Tertiary Hospital (n)** |
| --- | --- | --- |
| **Beijing** | 4 | 12 |
| **Chengdu** | 2 | 9 |
| **Guangzhou** | 0 | 11 |
| **Hangzhou** | 0 | 12 |
| **Shanghai** | 5 | 16 |
| **Shenyang** | 0 | 11 |
| **Tianjin** | 1 | 7 |
| **Zhengzhou** | 1 | 10 |
