# Supplementary material for: Prevalence and influencing factors of probiotic usage among colorectal cancer patients in China: A national database study
Source: PLoS One. 2023 Sep 21;18(9):e0291864. doi: 10.1371/journal.pone.0291864 (PMC10513277; doi:10.1371/journal.pone.0291864)
Supplement: S2 Table — (DOCX) [file pone.0291864.s002.docx]

**Supplementary table 2. Number of prescriptions from different hospitals (n= 101)**

| **Hospitals** | **Total CRC presciption number** | **CRC presciption number per day** |
| --- | --- | --- |
| **HC1** | 105986 | 378.5 |
| **HC2** | 82538 | 294.8 |
| **HC3** | 73354 | 262.0 |
| **HC4** | 58899 | 210.4 |
| **HC5** | 58599 | 209.3 |
| **HC6** | 52496 | 187.5 |
| **HC7** | 50282 | 179.6 |
| **HC8** | 47535 | 169.8 |
| **HC9** | 47160 | 168.4 |
| **HC10** | 45860 | 163.8 |
| **HC11** | 41002 | 146.4 |
| **HC12** | 37867 | 135.2 |
| **HC13** | 34909 | 124.7 |
| **HC14** | 34770 | 124.2 |
| **HC15** | 30364 | 108.4 |
| **HC16** | 19113 | 68.3 |
| **HC17** | 17008 | 60.7 |
| **HC18** | 15957 | 57.0 |
| **HC19** | 15853 | 56.6 |
| **HC20** | 15312 | 54.7 |
| **HC21** | 15274 | 54.6 |
| **HC22** | 15215 | 54.3 |
| **HC23** | 13732 | 49.0 |
| **HC24** | 13570 | 48.5 |
| **HC25** | 12936 | 46.2 |
| **HC26** | 12030 | 43.0 |
| **HC27** | 11896 | 42.5 |
| **HC28** | 11243 | 40.2 |
| **HC29** | 11180 | 39.9 |
| **HC30** | 9399 | 33.6 |
| **HC31** | 8477 | 30.3 |
| **HC32** | 7096 | 25.3 |
| **HC33** | 6875 | 24.6 |
| **HC34** | 6862 | 24.5 |
| **HC35** | 6683 | 23.9 |
| **HC36** | 6649 | 23.7 |
| **HC37** | 6577 | 23.5 |
| **HC38** | 6403 | 22.9 |
| **HC39** | 6380 | 22.8 |
| **HC40** | 6250 | 22.3 |
| **HC41** | 6155 | 22.0 |
| **HC42** | 5799 | 20.7 |
| **HC43** | 5779 | 20.6 |
| **HC44** | 5352 | 19.1 |
| **HC45** | 5196 | 18.6 |
| **HC46** | 5185 | 18.5 |
| **HC47** | 5137 | 18.3 |
| **HC48** | 4966 | 17.7 |
| **HC49** | 4859 | 17.4 |
| **HC50** | 4844 | 17.3 |
| **HC51** | 4552 | 16.3 |
| **HC52** | 4286 | 15.3 |
| **HC53** | 4207 | 15.0 |
| **HC54** | 3839 | 13.7 |
| **HC55** | 3734 | 13.3 |
| **HC56** | 3691 | 13.2 |
| **HC57** | 3635 | 13.0 |
| **HC58** | 3549 | 12.7 |
| **HC59** | 3193 | 11.4 |
| **HC60** | 3132 | 11.2 |
| **HC61** | 3080 | 11.0 |
| **HC62** | 2911 | 10.4 |
| **HC63** | 2757 | 9.8 |
| **HC64** | 2628 | 9.4 |
| **HC65** | 2597 | 9.3 |
| **HC66** | 2375 | 8.5 |
| **HC67** | 2296 | 8.2 |
| **HC68** | 2148 | 7.7 |
| **HC69** | 2104 | 7.5 |
| **HC70** | 2086 | 7.5 |
| **HC71** | 1948 | 7.0 |
| **HC72** | 1923 | 6.9 |
| **HC73** | 1586 | 5.7 |
| **HC74** | 1580 | 5.6 |
| **HC75** | 1506 | 5.4 |
| **HC76** | 1445 | 5.2 |
| **HC77** | 1365 | 4.9 |
| **HC78** | 1309 | 4.7 |
| **HC79** | 1132 | 4.0 |
| **HC80** | 999 | 3.6 |
| **HC81** | 782 | 2.8 |
| **HC82** | 737 | 2.6 |
| **HC83** | 543 | 1.9 |
| **HC84** | 538 | 1.9 |
| **HC85** | 527 | 1.9 |
| **HC86** | 441 | 1.6 |
| **HC87** | 428 | 1.5 |
| **HC88** | 366 | 1.3 |
| **HC89** | 313 | 1.1 |
| **HC90** | 302 | 1.1 |
| **HC91** | 268 | 1.0 |
| **HC92** | 153 | 0.5 |
| **HC93** | 109 | 0.4 |
| **HC94** | 101 | 0.4 |
| **HC95** | 100 | 0.4 |
| **HC96** | 73 | 0.3 |
| **HC97** | 39 | 0.1 |
| **HC98** | 19 | 0.1 |
| **HC99** | 10 | 0.0 |
| **HC100** | 1 | 0.0 |
| **HC101** | 1 | 0.0 |
