# Supplementary material for: Prevalence and influencing factors of probiotic usage among colorectal cancer patients in China: A national database study
Source: PLoS One. 2023 Sep 21;18(9):e0291864. doi: 10.1371/journal.pone.0291864 (PMC10513277; doi:10.1371/journal.pone.0291864)
Supplement: S3 Table — (DOCX) [file pone.0291864.s003.docx]

**Supplementary table 3. Formulations of probiotics**

| **Number of probiotic strains** | **Formulation** | **CFU^a^ per dosage unit** | **RDD^b^** | **Year of first authorisation** | **Storage temperature (**℃) | **Seven year usage (%)^c^** |
| --- | --- | --- | --- | --- | --- | --- |
| **Single** | *Bacillus coagulans* | 1.75×10^7^ | 9 | 2005 | 10-30 | 0.4 |
|  | *Bacillus cereus* | 2×10^8^ | 6 | 1997 | 2-25 | 0.1 |
|  | *Bacillus licheniformis* | 2.5×10^8^ | 6 | 1995; 2008 | 10-30 | 7.6 |
|  |  | 5×10^8^ | 3 | 2007 | 10-30 | 0.1 |
|  | *Bifidobacterium adolescentis* | 5×10^7^ | 3 | 1996 | 2-8 | 3.0 |
|  | *Clostridium butyricum* | 6.3×10^6^ | 6 | 2004 | 10-30 | 1.1 |
|  |  | 3.5×10^5^ | 6 | 2014 | 10-30 | 12.9 |
|  |  | 1×10^7^ | 6 | 2004; 2014 | 10-30 | 0.2 |
|  |  | 1×10^6^ | 3 | 2004 | 10-30 | 0.1 |
|  | *Saccharomyces boulardii* | 3.25×10^8^ | 1-4 | 2015 | below 25 | 0.1 |
| **Two** | *Enterococcus faecium*  *Bacillus subtilis* | 1.35×10^8^, 1.5×10^7^ | 1-4 | 2002 | below 20 | 0.0 |
|  |  | 4.5×10^8^, 5×10^7^ | 4 | 2003 | 10-30 | 6.7 |
|  | *Clostridium butyricum*  *Bifidobacterium infantis* | 4.2×10^6^, 4.2×10^5^ | 6 | 2002 | 2-8 | 4.8 |
|  |  | 5×10^6^, 5×10^5^ | 6 | 2002 | 2-8 | 0.4 |
| **Three** | *Clostridium butyricum*  *Bacillus mesentericus*  *Enterococcus faecalis* | 1×10^5^~1×10^8^, 1×10^5^~1×10^8^, 2×10^5^~4×10^8^ | 6 | 2008 | 10-30 | 2.0 |
|  | *Lactobacillus acidophilus*  *Enterococcus faecalis*  *Bacillus subtilis* | 5×10^6^ in total | 3 or 6 | 1994 | below 20 | 31.3 |
|  | *Bifidobacterium longum*  *Lactobacillus acidophilus*  *Enterococcus faecalis* | 1.0×10^6^ each | 6 | 1999 | 2-10 | 3.2 |
|  |  | 1×10^7^ each | 6 | 1995 | 2-8 | 13.7 |
|  | *Bifidobacterium longum*  *Lactobacillus bulgaricus*  *Streptococcus thermophilus* | 5×10^6^,  5×10^5^,  5×10^5^ | 10 | 1998 | 2-8 | 10.7 |
| **Four** | *Bifidobacterium infantis*  *Lactobacillus acidophilus*  *Enterococcus faecalis*  *Bacillus cereus* | 5×10^5^ each | 9 | 2006 | 2-8 | 1.6 |

^a^CFU, colony-forming units. ^b^RDD, recommended daily dose. RDD is according to the manufacturer's recommended dosage as the number of tablets, caplets, or other dosage units for adults. ^c^The five-year usage percentage is the product dosage divided by the sum of all products. The product dosage is the total number of units used for five years divided by the max RDD.
